# Cigarette smoke impairs granulosa cell proliferation and oocyte growth after exposure cessation in young Swiss mice: an experimental study

**DOI:** 10.1186/1757-2215-5-25

**Published:** 2012-09-20

**Authors:** Larissa LO Paixão, Rejane P Gaspar-Reis, Gabrielle PL Gonzalez, Aline S Santos, Aluana C Santana, Rachel MM Santos, Poli Mara Spritzer, Celly Cristina A Nascimento-Saba

**Affiliations:** 1 Department of Physiological Sciences, Institute of Biology Roberto Alcântara Gomes, University of the State of Rio de Janeiro, Av. 28 de Setembro, 87 PAPC. Vila Isabel, 20551-030 , Rio de Janeiro, Brazil; 2 Department of Physiology and Pharmacology, Biomedical Institute, Fluminense Federal University, Rua Professor Hernani Melo, 101. São Domingos, Niterói 24210-130, Niterói, Brazil; 3 Gynecological Endocrinology Unit, Division of Endocrinology, Hospital de Clinicas de Porto Alegre and Department of Physiology, Federal University of Rio Grande do Sul, Rua Ramiro Barcelos 2350, 90035-003, Porto Alegre, Brazil

**Keywords:** Tobacco, Cigarette smoking, Granulosa cell, Oocyte, Folliculogenesis, Female mice, Ovary, Morphometric aspects, Antral follicles, Smoke cessation

## Abstract

**Background:**

Cigarette smoke is associated with decreased female fertility, causing damage to ovarian function and disturbing follicle development. However, the effects of cigarette toxicants on ovarian function depend on duration and intensity of exposure. The aim of this study was to assess the effects of brief, intense exposure to tobacco smoke on granulosa cell number, oocyte growth, and follicle size during puberty in female Swiss mice.

**Methods:**

Ten female Swiss mice aged 35 days were exposed to tobacco smoke from 3R4F reference research cigarettes. They were exposed to an automatic smoking machine 8 h/day, 7 days/week for 15 days. Ten age-matched controls were kept in a different room and exposed to ambient air. At the end of 15 days, five mice in each group were euthanized and the ovaries were analyzed for follicular morphometry and granulosa cell count. The remaining animals were kept for an additional 30 days for further analysis as an ex-smoker group and control group. Comparison between the two groups was evaluated by the Student’s *t*-test or a two-way ANOVA followed by Bonferroni post-test was applied for multiple comparisons.

**Results:**

We found that cigarette smoke impaired antral follicular growth even after exposure cessation (p < 0.001). Both smoking and ex-smoking groups exhibited similar follicle diameter. However, at the same follicular stage, the number of granulosa cells was smaller in the ex-smoking group compared to smoking animals (p < 0.001). This was associated with increased oocyte diameter in ex-smoking animals compared to smoking animals (p < 0.01).

**Conclusions:**

The negative effects of cigarette smoking seem to last even after exposure has been interrupted. Moreover, brief exposure during puberty may induce silent oocyte disruption, which could in turn lead to decreased fecundity rates.

## Background

The prevalence of smoking among women of reproductive age has increased worldwide over the last years [1,2]. Smoking has a multitude of deleterious health effects and, especially, toxic effects on female reproduction [3]. There is evidence that 90% of smokers start this behavior during adolescence [4], and young women are the fastest growing population of smokers.

Smoke exposure is potentially damaging to ovarian function, affecting estradiol secretion and follicle development [5,6]. However, the mechanisms underlying the association between impairment of follicular development and smoking are not well known. Studies in mice have shown that damage to follicles occurs in early stages of development [7,8] and may be caused by low levels of anti-müllerian hormone and changes in the pro-oxidant/antioxidant balance [8,9]. Also, evidence suggests additional impairment in preovulatory follicles and oocyte maturation that alters ovulation rate [10,11].

It is well known that the effects of cigarette toxicants on ovarian function depend on duration, intensity, and type of exposure [7-11]. In addition, while cigarette smoking is related to various ovarian morphological alterations [3], the focus on follicular development is important because this is the first stage of maturation of female germ cells.

In spite of the evidence of harmful effects of cigarette smoking on folliculogenesis, no study so far has investigated the consequences of short-duration exposure to smoking during puberty. Therefore, the aim of this study was to assess the effects of brief, intense exposure to tobacco smoke on granulosa cell number, oocyte growth, and follicle size during puberty in female Swiss mice.

## Methods

### Animals

Twenty female Swiss mice were obtained after birth. Weanlings from different litters at 21 days of age were housed two or three per cage and allowed free access to commercial rodent chow (Nuvilab, Nuvital, Curitiba-Paraná, Brazil) and drinking water throughout the experiments. Visual inspection of the vulva was performed until detection of vaginal opening. Following vaginal opening, the mice were checked for estrous cyclicity by vaginal cytology at 9:00–10:00 am daily throughout the study. Only animals in the estrous phase were euthanized.

The study protocol and all procedures involving the experimental animals were in compliance with the principles of laboratory animal care [12] and national laws for use of laboratory animals. The study was approved by the Ethics Committee of the Biology Institute of the State University of Rio de Janeiro.

### Cigarette smoke exposure

Ten animals aged 35 days were exposed to tobacco smoke from 3R4F reference research cigarettes (University of Kentucky, Lexington, KY, USA) (nicotine = 0.73 mg/cigarette) for 15 days. Whole body exposure to tobacco smoke lasted 8 h/day, from 8 a.m. to 4 p.m., 7 days/week in a chamber that received smoke generated from an automatic cigarette smoking machine as previously described [13]. Ten age-matched controls (C1 group) were kept in a different room and exposed to ambient air. These animals had no contact with cigarette smoke at any time.

### Procedures

On the last day of exposure, five females in each group were euthanized using intraperitoneal tribromoethanol (Avertin®, Sigma-Aldrich, USA). Blood samples were obtained by cardiac puncture for cotinine quantification. Ovarian tissue obtained at necropsy was weighed and fixed in 10% formalin. The right ovary from each animal was used for the histological studies. The remaining animals formed two new groups: cigarette exposure animals were placed in a former ex-smoking group and non-exposed animals remained as age-matched controls (C2 group). At that stage, all females were maintained under the same conditions in an animal facility for 30 days with no exposure to cigarette smoke.

### Cotinine serum levels

Blood was centrifuged for 10 min at 3000 × g at 4°C, and serum was stored at −20°C. Cotinine levels were determined using a cotinine assay kit (Elisa, Orasure Technologies, Bethlehem, PA, USA), in accordance with the manufacturer’s recommendations.

### Histology and morphometric analysis of follicle and oocyte

Ovaries were fixed in formalin, dehydrated in ethanol and paraffin- embedded.

Serial sections of 5 μm thickness were prepared and stained with hematoxylin-eosin.

The identification of follicles within the serial sections was based on strict criteria. Follicles were classified according to Pedersen and Peters [14] into primary, preantral and incipient antral stages, considering animals in the estrous phase. Primary follicles presented a simple layer of cuboid granulosa cells, while preantral follicles had two or more granulosa cell layers. Incipient antral follicles were recognized by the presence of a visible antral cavity between granulosa cells.

All slides available for each ovary were examined to identify sections containing primary, preantral and incipient antral follicles. Five slides containing primary and another five containing preantral follicles were randomly chosen for analysis among those with the largest cross-area and analyzed. Regarding incipient antral follicles, five slides were randomly chosen for each ovary among those presenting both oocyte nuclei and vacuoles. The analysis was carried out by one trained investigator (LLOP), who was not blinded to treatment group.

For the morphometric evaluation, follicular diameters were determined as the average of two measurements at a right angle across the midpoints. In primary follicles, the diameter was measured from the outer layer of granulosa cells, and from the outer wall of the thecal layer in the preantral and incipient stages. Oocyte diameter was measured including the zona pellucida (15).

All diameters were measured using the Image-Pro Plus 4.5 software, after calibration with a stage micrometer (Media Cybernetics, Maryland, USA). Nuclei of granulosa cells were manually counted in images displayed in Adobe Photoshop® 7.0 (Adobe Systems Incorporated, San Jose, California).

### Statistics

Data were expressed as means and standard error of mean (SEM). Group comparisons were evaluated by the Student’s *t* test or two-way ANOVA followed by Bonferroni post-hoc test for multiple comparisons. Results were considered to be significant at p < 0.05.

## Results

### Conitine levels

Mice exposed to cigarette smoke for 15 days had mean serum cotinine concentrations similar to that found in exposed humans [7]. Exposure was significantly higher than that of controls (109.7 ± 19.4 ng/ml vs. 0.7 ± 0.7 ng/m, p = 0.05). Control mice showed cotinine levels below the limit of the technique (8 ng/ml).

### Morphometric analysis of ovarian follicles

**Figure 1:**
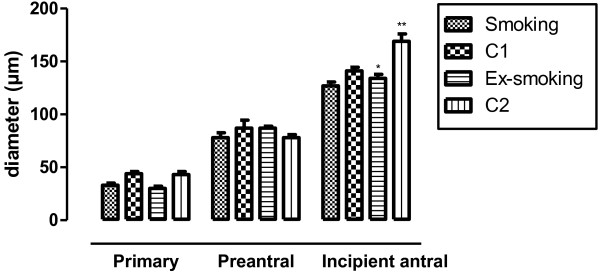
**Follicular diameter according to follicular stage.** Values are presented as mean ± SEM (n = 5). *p < 0.001 versus C2; **p < 0.001 versus C1.

Figure 1 shows follicular diameters stratified according to follicular stage classification. The diameter of primary and preantral follicles was similar in all four groups. However, the diameter of incipient antral stage follicles was larger in C2 than in the C1 and ex-smoking groups (p < 0.001).

**Figure 2:**
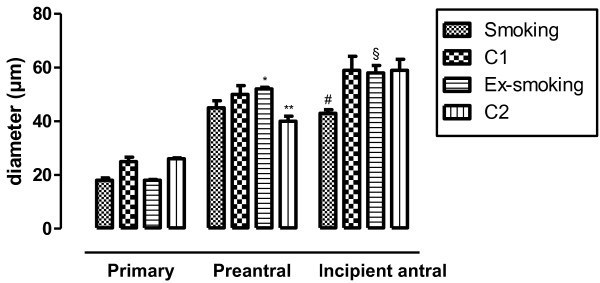
**Oocyte diameter according to follicular stage.** Values are presented as mean ± SEM (n = 5). * p < 0.05 versus C2; ** p < 0.05 versus C1; # p < 0.01 versus C1; § p < 0.01 versus smoking.

Oocyte diameter stratified according to follicular stage is shown in Figure 2. C2 mice had smaller oocyte diameter at the preantral follicle stage than ex-smoking and C1 mice (p < 0.01). Concerning incipient antral follicles, oocyte diameter of smoking animals was smaller when compared to C1 (p < 0.01). In addition, ex-smoking animals showed an increase in oocyte diameter compared to smoking animals (p < 0.01). No differences were observed between the groups in oocyte diameter in primary follicles.

**Figure 3:**
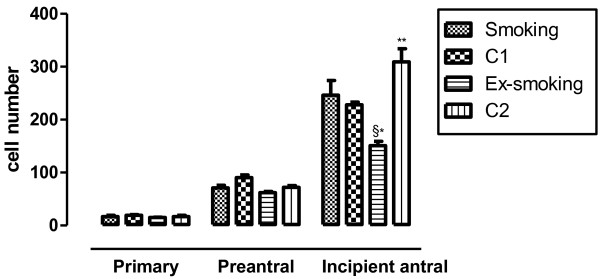
**Granulosa cell count according to follicular stage.** Values are presented as mean ± SEM (n = 5). § p < 0.001 versus smoking; *p < 0.001 versus C2; **p < 0.001 versus C1.

Granulosa cell count is presented in Figure 3. There were no differences between the groups in granulosa cell number in primary and preantral follicle stages. In contrast, at incipient antral follicle stage, a decrease was observed in granulosa cell number in the ex-smoking group compared to smoking animals (p < 0.001). Moreover, granulosa cell count was higher in C2 than in ex-smoking and C1 mice (p < 0.001).

### Estrous cycle and ovarian weight

Both ovarian weight and estrous cycle frequency were similar among groups (Table 1).

**Table 1:** Effects of smoke exposure on estrous cycle and ovarian weight

**Groups**	**No. of cycles**	**Proestrus**	**Estrus**	**Metestrus**	**Diestrus**	**Ovarian weight**
Smoking	2.8 ± 0.25	4.3 ± 0.55	8.8 ± 1.1	3.8 ± 0.68	1.7 ± 0.56	16 ± 2.4
C1	2.8 ± 0.36	4.6 ± 0.58	9.3 ± 0.86	4.9 ± 0.89	3.6 ± 0.71	12 ± 2.3
*Interval of 30 days*						
Ex-smoking	7.8 ± 0.97	16 ± 2.9	18 ± 1.4	14 ± 2.9	2.4 ± 1.3	19 ± 4.4
C2	8.2 ± 0.86	13 ± 2.0	21 ± 3.0	11 ± 1.6	4.4 ± 0.68	20 ± 3.2

Number of cycles and duration of estrous stage (days). Ovarian weight is presented as mg/100 g body weight. Smoking: smoke-exposed animals; C1: age-matched controls for smoking animals; Ex-smoking: animals carried on to cigarette smoke-cessation group; C2: age-matched controls for cigarette smoke-cessation mice. Comparison between groups by the Student’s *t*-test. Values are mean and SEM. No significant differences between smoking vs. C1 or ex-smoking vs C2.

## Discussion

The present study was designed to examine whether brief, intense tobacco exposure during puberty could impair granulosa cell and oocyte growth in mice in an experimental setup that accurately mimicked human exposure. We found that cigarette smoke impaired oocyte growth in incipient antral follicles of exposed mice. In addition, granulosa cell count did not increase with time in ex-smoking mice as occurred with controls. The number of granulosa cells is associated with normal oocyte development, and proliferation of these cells is also influenced by germ cells through cross-talk [15,16]. Thus, our results suggest that smoke exposure interfered negatively with oocyte development, and that this negative interference was associated with inadequate granulosa cell proliferation after smoke exposure cessation. In fact, elements such as cadmium found in cigarette smoke seem to impair granulosa cell proliferation [17,18]. Moreover, it may disrupt gap junction coupling between the oocyte and granulosa cells by increasing zona pellucida thickness [19] and consequently deranging the cross-talk between these cells.

The experimental interval of nicotine exposure corresponded to the approximate duration of pubertal and adolescent characteristics in most animals [20]. Because our aim was to study the impact of brief tobacco exposure specifically during puberty, we considered an interval starting at 35 and finishing at 50 days of age. Moreover, considering the study design, according to which we obtained data on ovarian morphology once during nicotine exposure and again 30 days after exposure cessation, we performed morphometric analyses of follicles from different stages on these two occasions, rather than following the growth and development of follicles.

Concerning cotinine levels as a biomarker for tobacco exposure, the high levels observed in our animals at the end of exposure indicate the effectiveness of the smoke chamber in delivering cigarette smoke to these animals. While the amount of inhaled cigarette smoke in rodents cannot be directly transferred to humans because of differences in nicotine catabolism, the cotinine levels found in exposed animals were similar to those reported by Caraballo et al. [21]. In addition, even though not directly measured 30 days after removal from the smoke chamber, we assumed that, in this experimental model, nicotine levels would no longer be detectable after 30 days as shown in a previous study in which nicotine concentrations were undetectable in mice exposed to air in an identical chamber [13].

Despite the finding of a smaller oocyte diameter in incipient antral follicles under nicotine exposure, an increase in oocyte size was observed after smoking cessation. While the present study was not designed to assess oocyte growth, it is well known [15] that, at incipient follicular stage, oocytes have already reached maximum growth and acquired competence to resume meiosis. However, a previous study [22] has reported that cigarette smoke could impair earlier oocyte maturation, and that reduced size may be regarded as a characteristics of immature oocytes. Therefore, our data are in agreement with the notion that smoke exposure could be associated with smaller germ cell diameter at exposure time. Additionally, as oocytes have an efficient DNA repair system [23], it is possible that in the absence of cigarette toxicants, oocytes may recover from smoke-related DNA damage. In fact, even under nicotine administration [24], aneuploidy was not observed in mice oocytes.

Contrary to oocyte size, granulosa cell count was dramatically reduced at incipient antral stage 30 days after, but not during, the smoke exposure period. This suggests that the impairment caused by smoke in these cells is not immediately evident. Some cigarette toxicants, like cadmium, have a long biological half-life [22,25], and accumulate over time in ovaries. In addition to nicotine damage on granulosa cells, which has been reported as promoting cell apoptosis [26], cadmium causes reduction in granulosa cell numbers [17,18,25], an effect which may persist beyond the exposure to smoke and nicotine.

In addition, in the present study, the observed arrest in follicular growth at the incipient antral stage through time suggests that the toxic effect of smoking exposure on follicular function lingers after smoke cessation. Indeed, a previous study [6] reported that benzo-[a]-pyrene, a cigarette toxicant, may reduce follicular growth in different stages of development.

Interestingly, antral follicles in control animals at older age seemed to grow better than in controls at an earlier age. While this finding may appear surprising, it is important to note that our first experimental period took place during the transition between puberty and adulthood, when the first reproductive cycles are starting. In contrast, 30 days later, during the second experimental period, the mice had already become young adults, and had reached reproductive maturity.

Concerning ovarian function, no differences were found in estrous cycle or ovarian weight in our exposed mice during either smoking exposure or after smoking cessation in comparison to control mice. Other investigators [27] have reported ovarian weight reduction after 8 weeks of cigarette exposure. This may be explained, at least partially, by the different duration of exposure. The short-duration smoking exposure used in the present study seems to promote an initial follicular damage with a silent effect that lasts even in the absence of further exposure.

Interestingly, a decrease in oocyte diameter in preantral follicles was found in C2 group. However, this was not accompanied by other disturbances in follicle or oocyte diameter in other follicle stages of C2 mice, as occurred with smoking mice. Additionally, granulosa cell number was even higher in the incipient antral stage of C2 animals. We hypothesize that this isolated finding might be due to the follicle selection process, in which less mature oocytes remain in preantral follicles and are not carried on to growing antral follicles.

Limitations of the present study are the lack of data on fecundity rates of our experimental mice and the absence of molecular analyses such as gene and protein expression in granulosa cells and oocyte, which could be helpful in providing further mechanistic insight. However, to our knowledge, this is the first study to assess early and late effects of brief and intense smoke exposure on granulosa and oocyte development. The morphometric analyses were able to demonstrate an initial damage that may potentially impair normal oocyte development. Further studies are needed on growth factor gene expression in order to elucidate the molecular mechanisms involved in cigarette exposure impairment of granulosa cell proliferation, oocyte growth and follicular development, as described in the present study.

## Conclusions

Exposure to cigarette smoke was associated with impaired granulosa proliferation and faulty oocyte development. Data from the present study suggest that the negative effects of cigarette smoking last even after exposure has been interrupted. Moreover, short-duration exposure during puberty may induce silent oocyte modifications that could be associated with low fecundity rates.

## Competing interests

The authors have no competing interests to declare.

## Authors' contributions

LLOP: acquisition of data, analysis and interpretation of data, manuscript drafting and contributed to final review. RPGR: contributed to study design and performed the statistical analysis. GPLG, ASS and ACS: acquisition of data. PMS: contributed to analysis and interpretation of data and revised critically the manuscript for important intellectual content. RMMS and CCANS: conception and study design, analysis and interpretation of data, drafting manuscript. All authors read and approved the final manuscript.
